# PRL-3 exerts oncogenic functions in myeloid leukemia cells via aberrant dephosphorylation of stathmin and activation of STAT3 signaling

**DOI:** 10.18632/aging.102290

**Published:** 2019-09-23

**Authors:** Jianping Xu, Wei Wu, Yao Tang, Yanfeng Lin, Yan Xue, Jianda Hu, Donghong Lin

**Affiliations:** 1 Department of Laboratory Medicine, School of Medical Technology and Engineering, Fujian Medical University, Fuzhou 350004, Fujian, China; 2 Department of Laboratory Medicine, Quanzhou Medical College, Quanzhou 362011, Fujian, China; 3 Fujian Institute of Hematology, Fujian Medical University Union Hospital, Fuzhou 350001, Fujian, China

**Keywords:** PRL-3, stathmin, serine phosphorylation, STATs signaling, myeloid leukemia

## Abstract

PRL-3, an oncogenic dual-specificity phosphatase, is overexpressed in 50% of acute myeloid leukemia patients. Stathmin has been identified as a downstream target of PRL-3 in colorectal cancer. However, the correlation between PRL-3 and stathmin in myeloid leukemia is unclear. In this study, we revealed the positive correlation between PRL-3 and stathmin in myeloid leukemia. Knockdown of the *PRL-3* gene by shRNA reduced the expression of downstream stathmin, suppressed cell proliferation, induced G2/M arrest and cell apoptosis, and inhibited migration and invasion in myeloid leukemia cells. Moreover, our study was the first to provide evidence that silencing PRL-3 increased the phosphorylation level in Ser16, Ser25, Ser38, and Ser63 of stathmin, and in turn inhibited the STAT3 and STAT5 signaling in myeloid leukemia cells. This evidence points to a promoted role for PRL-3 in the progression of myeloid leukemia, and PRL-3 could be a possible new treatment target.

## INTRODUCTION

Phosphatase of regenerating liver-3 (PRL-3), a dual-specificity phosphatase, belongs to class I cysteine-based protein tyrosine phosphatases (PTPs). PRL-3 is encoded by the *PTP4A3* gene, which is located on chromosome 8q24.3 [1, 2]. In normal tissue, PRL-3 mRNA expression has been found in skeletal muscle, the pancreas and the heart, and at lower levels in hematopoietic cells [3, 4]. However, PRL-3 protein has not been detected in mature human tissues [3]. PRL-3 was first discovered to be specifically up-regulated in metastatic colorectal cancer (CRC) cells in 2001 [5]. Since then, overexpression of PRL-3 has been implicated in a wide range of solid tumors, including gastric, ovarian and lung [6, 7]. Other than in the solid tumors, PRL-3 is overexpressed in 50% of acute myeloid leukemia (AML)and 90% of multiple myeloma (MM)patients [8, 9]. Previous study indicates that PRL-3 is transcriptionally regulated by STAT3, and the STAT3/PRL-3 regulatory loop contributes to the pathogenesis of AML [10]. Diverse roles of PRL-3 in tumor progression, including cell proliferation, migration, invasion, angiogenesis and metastasis, have been highlighted in recent reports that emphasize the importance of PRL-3 in tumorigenesis [11, 12].

*Zheng* et al. find that stathmin is a downstream target of PRL-3 in CRC. Interaction between PRL-3 and stathmin leads to aberrant microtubule destabilization, which promotes the progression and metastasis of CRC [13]. Stathmin is known as a highly conserved cytosolic phosphoprotein, and it can increase the rate of mitosis through up-regulation of microtubule dynamics [14]. Regulation of microtubule dynamics via phosphorylation and dephosphorylation at stathmin serine sites is essential for orderly progression through cell cycle. There are four serine phosphorylation sites (Ser16, Ser25, Ser38, and Ser63) at stathmin. Ser16 is phosphorylated by protein kinase C (PKC), or Ca^2+^/calmodulin-dependent kinase II/IV. Ser 63 is phosphorylated by cAMP-dependent protein kinase A [15, 16]. Ser25 and Ser38 are targeted by mitogen-activated protein kinases (MAPKs) and cyclin-dependent kinases (CDKs), respectively [17, 18]. The abnormal phosphorylation of the four different serine sites can directly result in the abnormal function activity of stathmin, which is the malignant proliferation of cells. Furthermore, our previous study showed that stathmin is highly expressed in primary and relapsed AML patients, whereas its expression is decrease or undetectable in remission patients. Patients with low expression after complete remission have a risk of relapse [19]. However, knowledge about the correlation between PRL-3 and stathmin in myeloid leukemia is unclear.

In the current study, we investigated (1) the correlation between PRL-3 and stathmin in myeloid leukemia; (2) the biological behavior in myeloid leukemia cells after *PRL-3*-silencing; and (3) the roles of stathmin in PRL-3 mediated myeloid leukemia pathogenesis. Our findings demonstrate that targeting PRL-3/stathmin may be of clinical benefit in treatment of myeloid leukemia with high PRL-3 expression.

## RESULTS

### Correlation analysis

**Figure 1:**
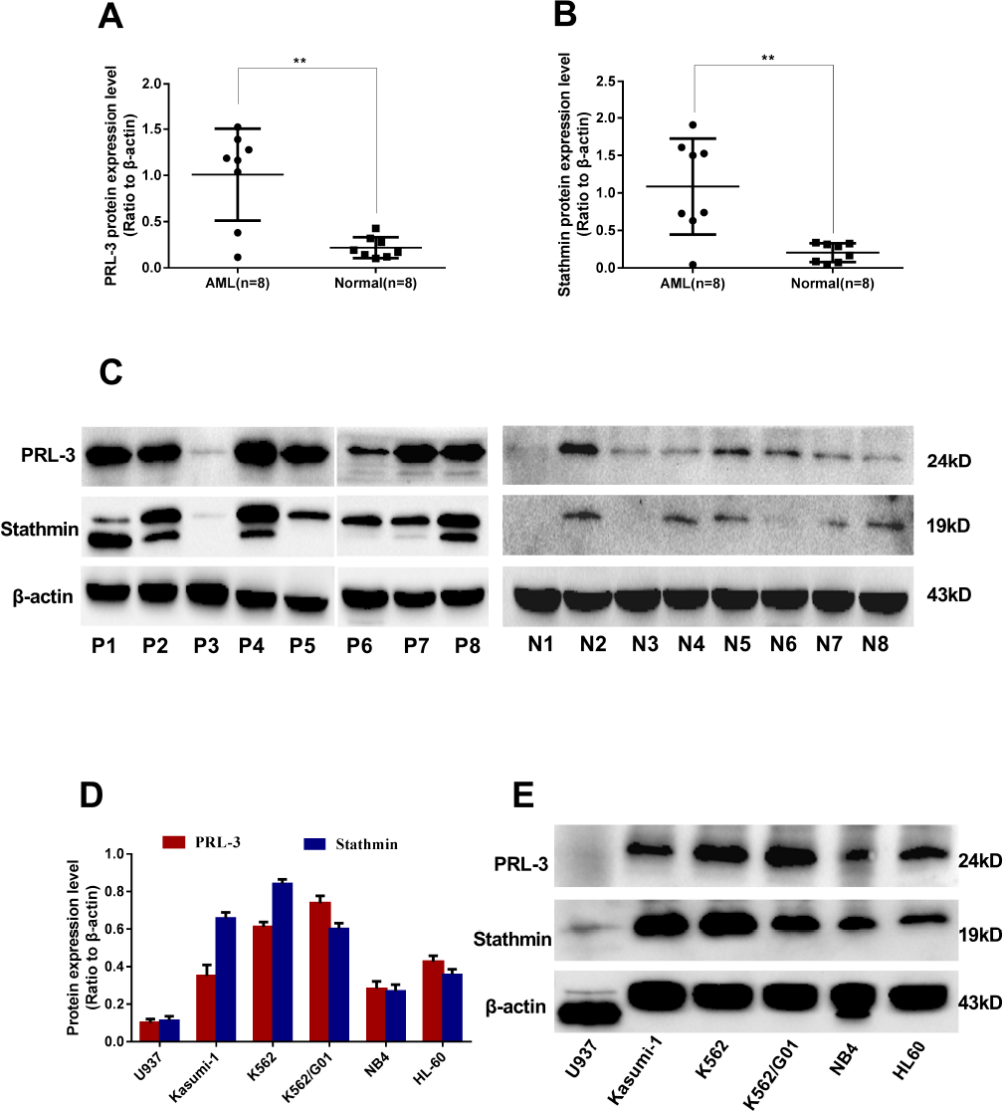
**Expression of PRL-3 and stathmin in clinical samples and cell lines detected by western blot.** (**A**–**C**) Expression of PRL-3 and stathmin in de novo myeloid leukemia patients. (**D**, **E**) Expression of PRL-3 and stathmin in myeloid leukemia cell lines. (*P<0.05, **P<0.01, *vs*. healthy normal control). *Note*: N, healthy normal control. P, de novo patient.

To study the relevance between PRL-3 and stathmin, western blot was used to detect the expression of PRL-3 and stathmin in clinical samples and cell lines. The median expression of PRL-3 and stathmin in de novo myeloid leukemia patients was 1.175 (Figure 1A) and 1.121 (Figure 1B), respectively. They were significantly higher than those of normal controls (*P*<0.01). The data demonstrated that both PRL-3 and stathmin were overexpressed in de novo myeloid leukemia patients (Figure 1C). Spearman correlation analysis revealed a positive correlation between them in clinical samples (r=0.623, *P*<0.01). Meanwhile we detected the expression of PRL-3 and stathmin in independent myeloid leukemia cell lines including HL-60, Kasumi-1, NB4, U937, K562, and imatinib resistant K562/G01 (Figure 1E). There was a significant positive correlation in these cell lines (r=0.709, *P*<0.01), which was consistent with the result of clinical samples. The mRNA expression of PRL-3 and stathmin in these cell lines had a similar trend with protein (data not shown). Owing to its higher expression of PRL-3 (Figure 1D), we used K562 and K562/G01 cells for our further study.

### *PRL-3*-silencing reduces the expression of stathmin in K562 cells

**Figure 2:**
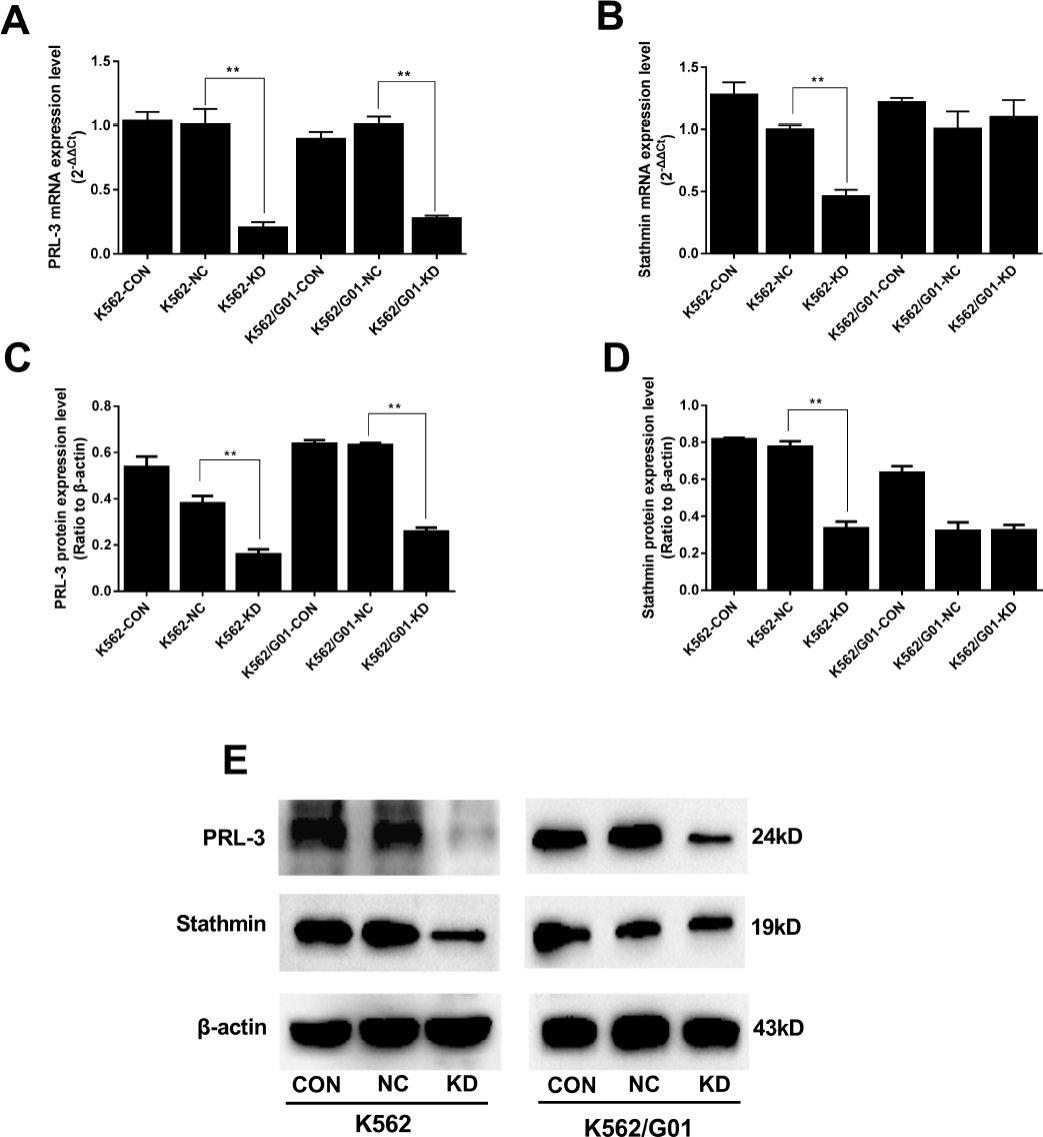
**Expression of PRL-3 and stathmin was assessed after *PRL-3*-silencing in K562 and K562/G01 cells.** (**A**) The mRNA expression of *PRL-3* after transfection. (**B**) The mRNA expression of s*tathmin* after *PRL-3*-silencing. (**C**, **D**) Quantification of PRL-3 and stathmin were normalized to β-actin. (**E**) Western blot of PRL-3 and stathmin expression were detected after shPRL-3. (*P<0.05, **P<0.01, *vs.* NC group).

Data from qPCR and western blot showed that PRL-3 expression in the KD groups was markedly decreased in both mRNA and protein (Figure 2A, 2C, 2E). We further examined the change of downstream stathmin in *PRL-3*-silencing K562 and K562/G01 cells. Down-regulation of PRL-3 dramatically reduced the mRNA and protein expression of stathmin in the K562-KD group, compared with the NC group (*P*<0.01). In contrast, stathmin remained unchanged in the K562/G01-KD group (Figure 2B, 2D, 2E).

### *PRL-3*-silencing inhibits proliferation and colony formation in K562 cells

**Figure 3:**
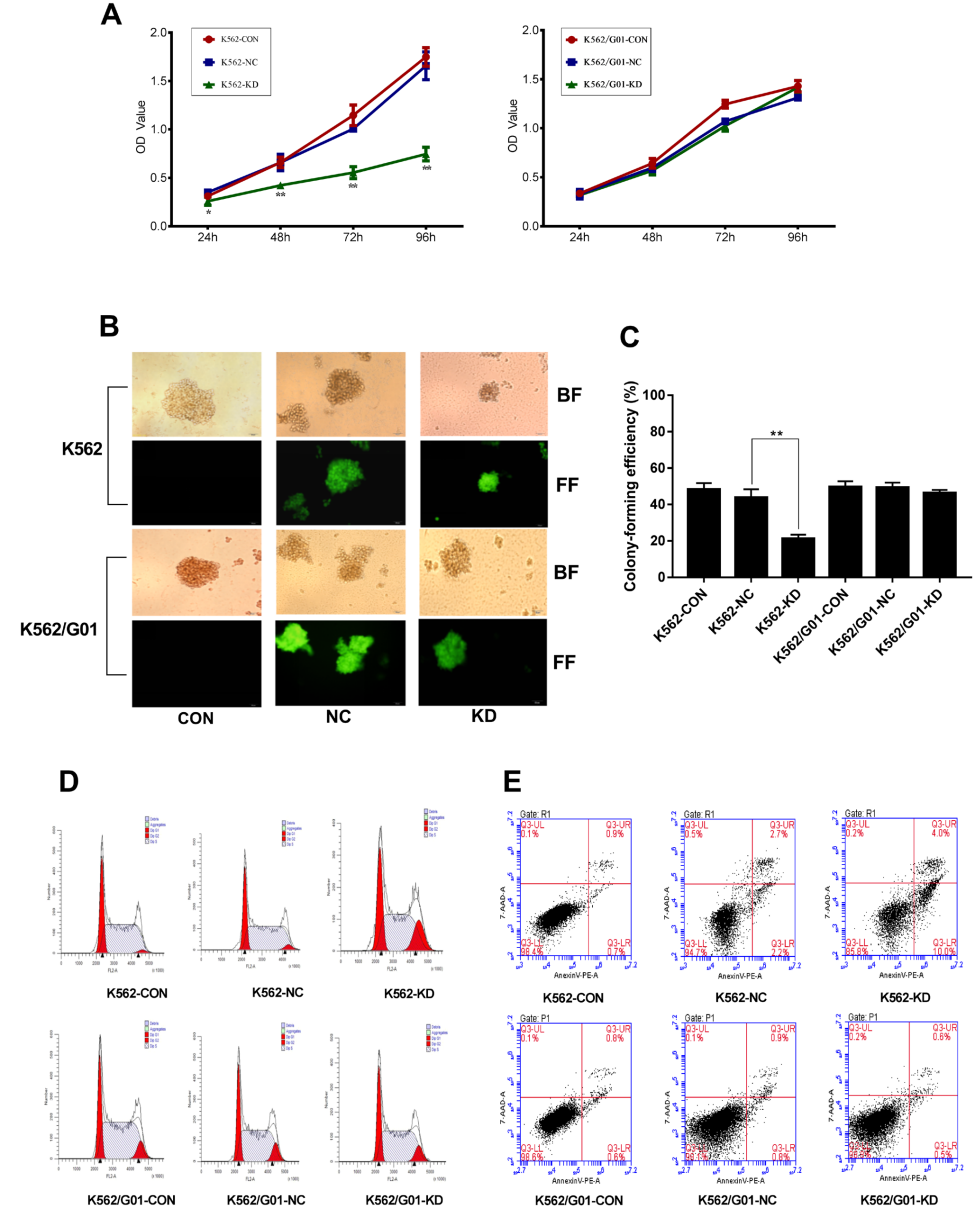
**Effects of *PRL-3*-silencing** **on cell proliferation and apoptosis were evaluated in K562 and K562/G01 cells.** (**A**) A cell growth curve was plotted based on the OD value (proportional to cell numbers) obtained at different time points following transfection. (**B**, **C**) Colonies containing ≥40 cells were counted on day 7 using a microscope (×200). (**D**) Cells were labeled by PI and analyzed using FCM. (**E**) Apoptotic cells were measured by FCM. Dot plots show 7-AAD (y-axis) vs. Annexin-V (x-axis). (*P<0.05, **P<0.01, *vs.* NC group).

To determine whether down-regulation of PRL-3 inhibited cell proliferation, our study examined cell growth and colony-forming abilities in K562 and K562/G01 cells. As shown in Figure 3A, knockdown of *PRL-3* by shRNA induced a time-dependent, progressive decrease in K562 cell viability. The OD value dropped from 1.006±0.031 to 0.554±0.062 (*P*<0.01), compared with the NC group at 72h following transfection. Concomitantly, shPRL-3 not only significantly increased the loss of clonogenic survival of the K562 (Figure 3C), but also decreased the size of the colonies (Figure 3B). While in K562/G01 cells, the OD value of the KD group and the NC group were statistically similar (*P*>0.05) (Figure 3A) at the same time point. And the ratio of colony formation of the KD group and the NC group was 46.250±1.750% and 49.250±2.750%, respectively (*P*>0.05) (Figure 3B, 3C).

### *PRL-3*-silencing induces G2/M phase arrest and apoptosis in K562 cells

To evaluate the effect of *PRL-3* gene silencing on the cell cycle distribution, cell cycle analysis was performed. As shown in Figure 3D, the percentage of G2/M phase in the K562-KD group was 17.86±1.673%, which was higher than that of the NC group (5.047±1.670%) (*P*<0.01). Additionally, the percentage in the S phase was significantly decreased in the K562-KD group (55.03±1.557%) compared with the NC group (69.17±2.715%) (*P*<0.01). The data suggested that down-regulation of PRL-3 in K562 cells resulted in cell cycle arrest in G2/M phase. We further conducted the apoptosis assay. As anticipated, the total apoptosis ratio of K562-KD group reached to 14.133±0.513% in comparison to the NC group (*P*<0.01) (Figure 3E). Moreover, the percentage of early apoptotic cells (Annexin-V PE^+^ 7AAD^−^) was 10.667±1.890% in the K562-KD group. However, *PRL-3*-silencing led to a modest change of G2/M phase in the K562/G01-KD group (*P* > 0.05) (Figure 3D). And the apoptosis assay showed that there was no significant difference in the K562/G01-KD group and NC group (*P* > 0.05) (Figure 3E).

### *PRL-3*-silencing suppresses migration and invasion in K562 cells

**Figure 4:**
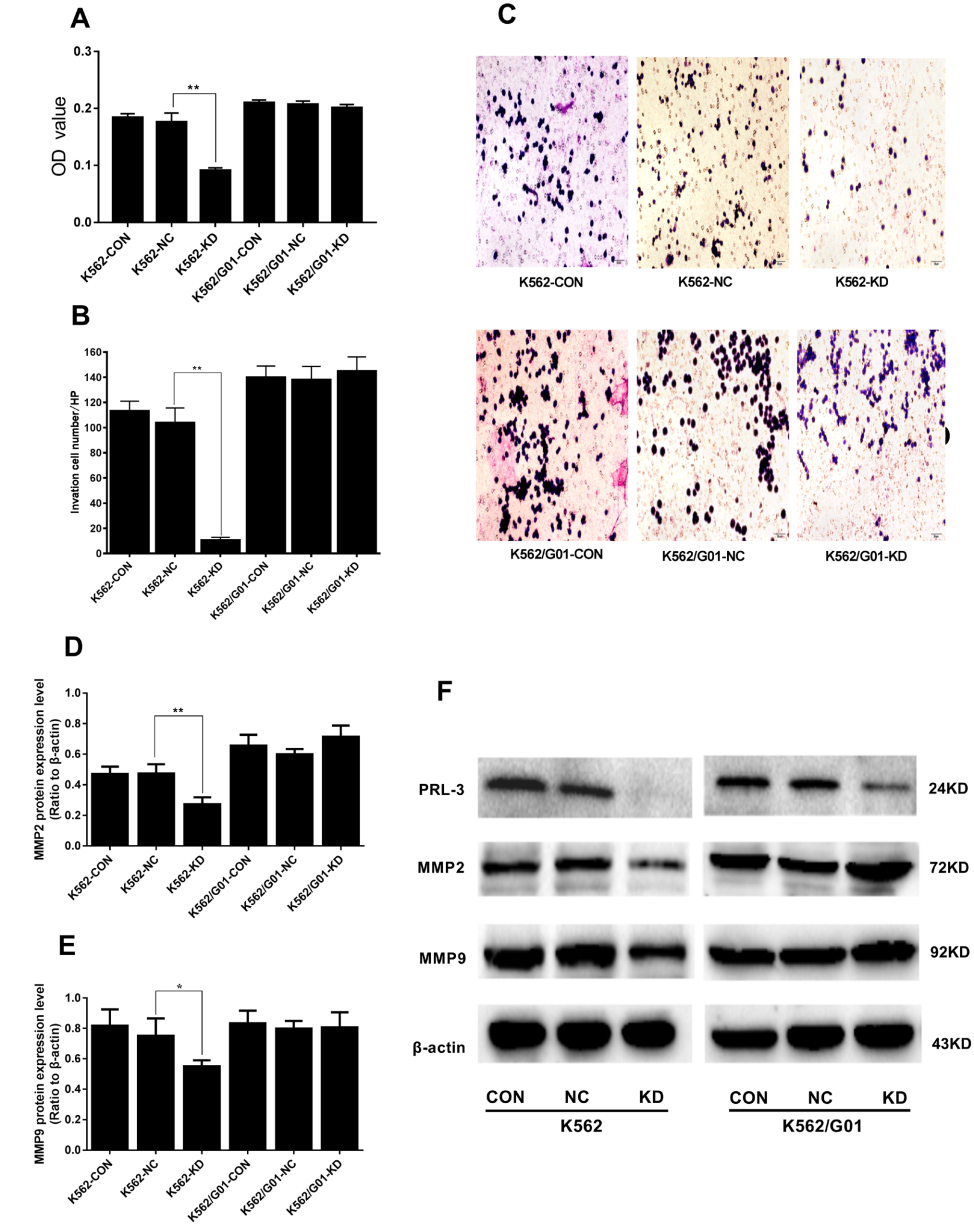
**Effects of *PRL-3*-silencing on cell migration and invasion were evaluated in K562 and K562/G01 cells**. (**A**) The OD values (proportional to cell numbers) of migrated cells were measured by MTS assay. (**B**) The invasion cell numbers were counted under microscope in five HP fields. (**C**) Wright-Giemsa stained invasion cells were observed under microscope (×200). (**D**, **E**) Quantification of MMP2 and MMP9 were normalized to β-actin. (**F**) MMP2, MMP9, PRL-3 and β-actin expression by western blot. (*P<0.05, **P<0.01, *vs.* NC group).

To investigate whether down-regulation of PRL-3 reduced the capacity of cell migration and invasion *in vitro*, a transwell chamber assay was performed. The OD value of migrated cells in the K562-KD group and the NC group was 0.091±0.005 and 0.176±0.016, respectively (*P*<0.01) (Figure 4A). And the migration inhibition ratio of the K562-KD group reached 48.295%. In parallel the invasion capacity dropped to 68.167% in the K562-KD group (*P*<0.01) (Figure 4B, 4C). Following, we measured the expression of migration and invasion related protein, including MMP-2 and MMP-9. Consistently, the expression of MMP2 (*P*<0.01) and MMP9 (*P*<0.05) in the K562-KD group were significantly decreased (Figure 4D–4F). No similar alternations in migration and invasion were observed in K562/G01 cells (P>0.05). Collectively, these data showed that down-regulation of PRL-3 could suppress the migration and invasion of K562 cells *in vitro*.

### *PRL-3*-silencing reduces dephosphorylation of stathmin and inhibits the activation of STAT3 and STAT5 signaling in K562 cells

**Figure 5:**
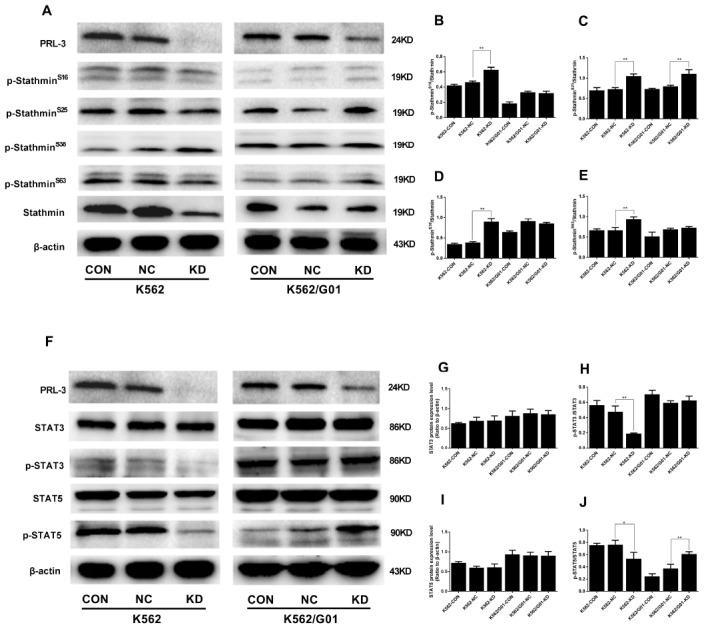
**The protein phosphorylation of stathmin and** **STATs signaling expressed in K562 and K562/G01 cells after *PRL-3*-silencing.** (**A**) Stathmin, the four stathmin-serine sites, and β-actin expression were detected by western blot. (**B**–**E**) Quantification of stathmin-phospho (Ser16, Ser25, Ser38, and Ser63) were normalized to stathmin. (**F**) STAT3, p-STAT3, STAT5, p-STAT5 and β-actin expression were detected by western blot. (**G**–**J**) Quantification of p-STAT3 and p-STAT5 were normalized to STAT3 and STAT5, respectively. (*P<0.05, **P<0.01, *vs.* NC group).

As mentioned above, phosphorylation and dephosphorylation of stathmin at four different serine sites could affect the cell cycle and cellular proliferation. Next we examined the phosphorylation of stathmin. Compared with the NC group, *PRL-3*-silencing dramatically increased the phosphorylation at Ser16, Ser25, Ser38, and Ser63 of stathmin in the K562-KD group (*P*<0.01) (Figure 5A–5E). Constitutive activation of JAK/STAT signaling pathway is frequently observed in leukemia cells [20]. Thus, we investigated whether PRL-3 influenced the activation of STATs signaling. shPRL-3 remarkably decreased the phosphorylation of STAT3 (*P*<0.01) (Figure 5H) and STAT5 (*P*<0.05) (Figure 5J) in the K562-KD group. Total STAT3 and STAT5 were unaffected by shPRL-3 (*P*>0.05) (Figure 5F, 5G, 5I). The results indicated that down-regulation of PRL-3 enhanced the phosphorylation of stathmin, which might inhibit the constitutive activation of STAT3 and STAT5 signaling in K562 cells.

Different results were observed in the K562/G01-KD group. The ratio of Ser25 phosphorylated protein in stathmin was significantly higher than that in the NC group (*P*<0.01), while other phosphorylated sites showed no significant difference (*P*>0.05) (Figure 5A–5E). To our surprise, we observed that p-STAT5 in the K562/G01-KD group was significantly higher than that in the NC group (*P*<0.01). At the same time, there was no alteration in p-STAT3 (Figure 5F–5J).

## DISCUSSION

In the present study, we revealed that there is a positive correlation between PRL-3 and stathmin in myeloid leukemia. Of note, we are the first to demonstrate that through the aberrant phosphorylation of the four serine sites, stathmin mediates the role of PRL-3 in myeloid leukemia progression via targeting STAT3 signaling.

Evidence suggests that PRL-3 has pro-oncogenic properties in AML. Elevated PRL-3 expression occurs in about 50% of AML patients while it is absent in normal myeloid cells from bone marrow [8]. In addition, a large-scale study of primary AML patients demonstrates that high level of PRL-3 is an independent negative prognostic factor in AML, both for overall survival and event-free survival [21]. *Zheng* et al. further find that stathmin, as a new downstream target of PRL-3 in SW480 cells, is decreased when PRL-3 is down-regulated. *Zheng* et al. therefore conclude that direct interaction between PRL-3 and stathmin cause abnormal microtubule depolymerization in colon cancer cells, and promote the cell cycle, which plays a critical role in the progression of CRC [13]. However, there are few reports on the correlation between PRL-3 and stathmin in myeloid leukemia. Therefore, in this study we analyzed the expression of PRL-3 and stathmin in myeloid leukemia patients and myeloid leukemia cell lines. Our data suggested that there is a positive correlation between PRL-3 and stathmin in myeloid leukemia. Thus we chose K562 and K562/G01 cells for our further study owing to the higher expression of PRL-3. Interestingly, a corresponding decrease in stathmin was observed with the down-regulation of PRL-3 in K562 cells. This result was similar with published work that shows the knockdown of *PRL-3* reducing the levels of stathmin in AML cells, Molm-14 and HEL cells [22].

To assess the roles of PRL-3 in pathogenesis of myeloid leukemia, further studies on the biological behavior were performed. With *PRL-3*-silencing, we found a clear reduction in cell viability and increased apoptosis in K562 cells. These results were in accordance with the study that shows significant effect of PRL-3 knockdown by siRNA on proliferation or apoptosis in classical Hodgkin lymphoma (HL) [23]. Cell cycle analysis indicated that *PRL-3*-silencing led to accumulation of K562 cells in the G2/M phase. These data implied that shPRL-3 inhibited proliferation through cell cycle blockage. To verify the speculation, we assessed the phosphorylation of stathmin at the four serine sites. Western blot results showed that the expression of stathmin was decreased by shPRL-3, meanwhile phosphorylation level of the four serine sites were markedly increased in K562 cells. As is known, stathmin is a downstream target of PRL-3 and plays a critical role in cell cycle progression [13]. The activity of stathmin is switched off at the onset of mitosis by phosphorylation to allow microtubule polymerization. Phosphorylated stathmin has to be reactivated by dephosphorylation upon entry into a new interphase [24]. That is, abnormal phosphorylation of stathmin led to the cell cycle blockage and induced cell apoptosis. It has been reported that inhibiting stathmin through different means result in cancer cell cycle arrest in G2/M phase and induce apoptosis [25, 26]. Together, this might be an explanation for inhibited proliferation and induced apoptosis in K562 shPRL-3.

It has been reported that migration and motility are linked to PRL-3 overexpression in several cancers [27–29]. PRL-3 can up-regulate the expression of MMP-2 and MMP-9, which promote peritoneal metastasis of gastric cancer cells [30]. Knockdown of PRL-3 in HL cell lines L1236 and HDLM2 reduces migration towards a CCL19 gradient [23]. In this study, shPRL-3 significantly reduced migration and invasion in K562 cells. It was consistent with the conclusion previously reported in HL. Furthermore, a recent study has shown that the blocking activation of STAT3 can reduce the expression of MMP2 and inhibit the invasion ability of cancer cells [31]. Meanwhile STAT3 can activate MMP9 in human dermal fibroblasts [32]. STAT3 can be constitutive activation by overexpression of PRL-3 in MM cells [33]. The activation of STAT3 and STAT5 directly promote transcription of PRL-3 in AML cells [10, 34]. Whether *PRL-3*-silencing affected the activation of STATs signaling was also addressed in this study. Our data suggested that the activation of STAT3 and STAT5 were remarkably suppressed by shPRL-3 in K562 cells. Based on the available data, down-regulation of PRL-3 could decrease the expression of stathmin and enhance the phosphorylation of stathmin. Since stathmin is a signal relay station, we speculated that abnormal phosphorylation of stathmin in turn inhibited the STAT3 and STAT5 signaling, which might partly reverse the malignant characteristics of K562 cells.

However, down-regulation of PRL-3 could not significantly reduce the expression of stathmin in K562/G01 cells. Furthermore, shPRL-3 had little effect on cell proliferation, cell cycle, cell apoptosis, and migration and invasion in K562/G01 cells. These results were beyond our anticipation. We merely observed that phosphorylation of the stathmin Ser 25 was markedly increased after *PRL-3*-silencing. PRL-3 as a dual-specificity phosphatase (DUSP), is also identified as a MAPK phosphatase (MKP-DUSP), and participates in the dephosphorylation of the MAPK signaling pathway [2]. Stathmin Ser25 targeted by MAPK was abnormally phosphorylated after *PRL-3*-silencing in K562 and K562/G01 cells. Our data indicated that stathmin Ser25 was an important regulation site by PRL-3 in myeloid leukemia cells. Remarkably, phosphorylation at either Ser 16 or Ser 63 strongly reduces the ability of stathmin to bind to and sequester soluble tubulin [35]. In comparison, phosphorylation at Ser 25 and/or Ser38 is not sufficient to inhibit the association between α/β-tubulin and stathmin. Hence, high expression of stathmin still maintained functional activity after *PRL-3*-silencing in K562/G01 cells.

There is still a problem. Why the K562 and K562/G01 cells react differently to shPRL-3 remains. As is known, erythroleukemia cell line K562 was derived from a patient with chronic myeloid leukemia (CML) in blast crisis phase [36]. Imatinib, as a first-generation tyrosine kinase inhibitor (TKI), is used for the first-line treatment of bcr/abl^+^ CML patients [37]. An imatinib-resistant human myeloid leukemia cell line K562/G01 was established in 2004. *Qi* et al. find that K562/G01 cells have increased levels of bcr/abl and the increased tyrosine kinase activity in comparison to K562 cells [38]. Bcr/abl can induce cellular transformation by activating the STAT5, and STAT5 is a critical factor for the sensitivity of CML progenitor cells to TKIs [39]. It has been reported that PRL-3 is a downstream target of bcr/abl signaling, and imatinib dose dependently decreased p-STAT3, p-STAT5 and PRL-3 in K562 and KCL-22 cells [40]. Interestingly, in our study the constitutive activation of STAT5 was observed in K562/G01 cells after shPRL-3, not in K562 cells. Owing to imatinib resistance in K562/G01 cells, we speculated that activation of STAT5 signaling might weaken the effect of PRL-3 to stathmin after *PRL-3*-scilencing. It might well explain the different reaction in K562 and K562/G01 cells after silencing *PRL-3* gene.

However, all of the data obtained were only based on the investigation in one myeloid leukemia cell line K562, so the data might not be convincing enough. Therefore, myeloid leukemia cell line Kasumi-1 with moderately expression of PRL-3 was also chosen to knock down the *PRL-3* gene by shRNA (data not shown). As expected, a corresponding decrease in stathmin was observed with the down-regulation of PRL-3. The following detection about the phosphorylation of stathmin and STATs signaling were performed in Kasumi-1 cells (data not shown). Western blot results were similar with the changes in K562 cells. The data of Kasumi-1 cell line are helpful to verify our opinion from molecular mechanism. Through the phosphorylation of the four serine sites, stathmin mediated the role of PRL-3 in myeloid leukemia progression via targeting STAT3 signaling. It is worthwhile to further investigate whether the small molecule inhibitors of PRL-3 exert similar effects as the shPRL-3, which will open up possibility for *in vivo* experiments.

**Figure 6:**
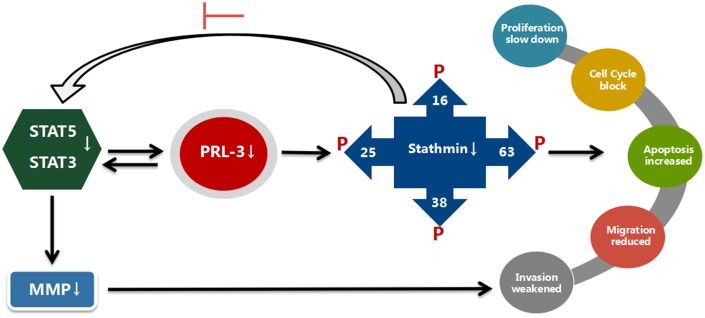
**Suggested mechanisms for a possible interplay between PRL-3, stathmin, and STATs.** With blocking of PRL-3, expression and activity of stathmin is reduced, and STAT3 and STAT5 activity are suppressed in K562 cells. The proliferation, migration, and invasion are reduced. In contrast, expression and activity of stathmin is not altered with shPRL-3 in K562/G01 cells, which results in K562/G01 cells maintaining malignant phenotypes.

In summary our current work suggests that PRL-3 and stathmin have a positive correlation in myeloid leukemia. shPRL-3 reduces the expression of downstream stathmin, suppresses proliferation and migration, and induces apoptosis. This process occurs probably by enhancing the phosphorylation of four stathmin serine sites, thus targeting suppression of STAT3 signaling (Figure 6). Of note, these findings indicate that PRL-3 may represent a novel target for treatment of myeloid leukemia.

## MATERIALS AND METHODS

### Cell lines and cell culture

Myeloid leukemia cell lines (HL-60, NB4, U937, and K562), imatinib resistant K562/G01 cell line were from CCTCC (China Center for Type Culture Collection, Wuhan, China). Myeloid leukemia cell line Kasumi-1 (gift from Prof. Ligen Liu, The Fifth People's Hospital of Shanghai, Fudan University, Shanghai, China). Cells were cultured in RPMI-1640 supplemented with 10% FBS. Imatinib (4 μM) was added to K562/G01 cell culture to maintain drug resistance. K562/G01 cells were grown in imatinib-free culture medium at least two weeks for each experiment.

### Clinical samples collection

The peripheral blood samples before chemotherapy were obtained from 8 newly diagnosed myeloid leukemia patients in Union Hospital of Fujian Medical University. The blast cells in peripheral blood were more than 80%. Normal controls from 8 healthy volunteers were also recruited. Peripheral blood mononuclear cells were isolated using density gradient centrifugation. All patients had given informed consent. The study was approved by the Ethics Committee in Fujian Medical University.

### Lentiviral transduction for PRL*-*3 knockdown

Short hairpin RNA (shRNA) was from Genechem Company (Shanghai, China). The shRNA targeting the *PRL-3* gene had the antisense: 5′-CCTGTTCTCGGCA CCTTAA-3′. Three groups were established, which included KD group (transfected with *PRL-3*-shRNA-LV), NC group (transfected with scramble-shRNA-LV) and CON group (blank control). Transfected cells were grown in medium containing 1.5 μg/ml puromycin for selection.

### Quantitative PCR analysis

Total RNA was extracted with TRIzol reagent and reverse transcribed into cDNA. qPCR was done using Power SYBR Green PCR Master Mix (TIANGEN, Beijing, China). Primer sequences of *GAPDH*, *PRL-3* and s*tathmin* for qPCR were the following: *PRL-3* Forward: 5′-GCTTCCTCATCACCCACAAC-3′, Reverse: 5′-ACTTC ACACACACGCACCAC-3′; s*tathmin* Forward: 5'-TCAG CCCTCGGTCAAAAGAAT-3′, Reverse: 5′-TTCTCGTG CTCTCGTTTCTCA-3′; *GAPDH* Forward: 5′-TCTCTGC TCCTCCTGTTC-3′, Reverse: 5′-GCCCAATACGACCA AATCC-3′. The comparative 2^-ΔΔCt^-method was used for relative quantification, with *GAPDH* as endogenous reference.

### Cell proliferation assay

Cells (0.25×10^5^) were seeded in 96-well plates, and cultured for 24h, 48h, 72h, and 96h. Cells were then pulsed with 20μl MTS and incubated at 37^°^C for 3h. The absorbance was measured at 492/630 nm using a Microplate Reader (MK3, Thermo Fisher Scientific, USA).

### Colony formation assay

Cells (4×10^2^) were plated in 24-well plates containing 0.8% methylcellulose (Sigma, CA, USA), and routinely cultured for 7-10 days. The number of colonies (containing ≥40 cells) was counted and the efficiency of colony formation was calculated.

### Cell cycle analysis

Cells (5.0×10^5^) were incubated with 100μl RNase A at 37^°^C for 30 min. Then cells were stained with PI (Keygen, Nanjing, China) at 4^°^C for 30 min and analyzed using a flow cytometry (Accuri C6, BD Bioscience, USA).

### Apoptosis assay

Cells were harvested and resuspended with binding buffer, then stained with Annexin V-PE/7-AAD (BD Bioscience, USA) according to the manufacturer’s instruction. The apoptotic cells were quantified by flow cytometry (Accuri C6, BD Bioscience, USA).

### Migration and invasion assay

The capacity of migration and invasion was assessed using Transwell plate with a membrane of pore size 8 μm (Coring, MA, USA). Matrigel was coated on the upper chamber for invasion assay according to the manufacturer’s protocol. Cells (1×10^5^) were seeded to the upper chamber. After 24h of incubated at 37°C, for migration assay: cells dropped to the lower chamber were detected using MTS. For invasion assay: cells attached to the lower surface of the membrane on upper chamber were fixed by methanol and stained with Wright-Giemsa. The upper chamber cells were counted using a microscope (IX71, Olympus, Japan).

### Western blot analysis

Cells were subjected to western blot analysis following a standard protocol. Antibodies against PRL-3, STAT3, p-STAT3 (Tyr705), STAT5, and p-STAT5 (Tyr694) were from Cell Signaling Technology (Beverly, MA, USA). Antibodies against stathmin and p-stathmin (S16, S25, S38, S63) were from Abcam (Cambridge, UK). Antibodies against MMP2, MMP9 and β-actin were from Santa Cruz Biotechnology (Dallas, TX, USA). β-actin was used as the internal reference. Quantification of the band densitometry was performed by Image J 1.43 software (NIH, MD, USA).

### Statistical analysis

Data are presented as mean ± standard deviation (SD) and analyzed base on SPSS 18.0 software. Students’t-test, or one-way analysis of variance (ANOVA) was performed to compare differences between groups. The Spearman method was performed to correlate analysis. A value of P < 0.05 was considered statistically significant.
